# *Antrodia cinnamomea* Confers Obesity Resistance and Restores Intestinal Barrier Integrity in Leptin-deficient Obese Mice

**DOI:** 10.3390/nu12030726

**Published:** 2020-03-10

**Authors:** Yi-Ting Tsai, Jhen-Wei Ruan, Cherng-Shyang Chang, Mei-Lan Ko, Hsiu-Chuan Chou, Chi-Chien Lin, Chiao-Mei Lin, Chih-Ting Huang, Yu-Shan Wei, En-Chi Liao, Hsin-Yi Chen, Cheng-Yuan Kao, Hong-Lin Chan

**Affiliations:** 1 Institute of Bioinformatics and Structural Biology, National Tsing Hua University, Hsinchu 30013, Taiwan; peach19920722@gmail.com (Y.-T.T.); t91050127@hotmail.com.tw (Y.-S.W.); nakyla1215@gmail.com (E.-C.L.); dful690@yahoo.com.tw (H.-Y.C.); 2 Department of Medical Laboratory Science and Biotechnology, National Cheng Kung University, Tainan 70101, Taiwan; jhenweiruan@mail.ncku.edu.tw; 3 Immunology Research Center, National Health Research Institutes, Zhunan, Miaoli 35053, Taiwan; rebear0330@gmail.com (C.-S.C.); chiaomei@nhri.edu.tw (C.-M.L.); cindyhuang@nhri.edu.tw (C.-T.H.); 4 Department of Ophthalmology, National Taiwan University Hospital Hsin-Chu Branch, Hsinchu 30059, Taiwan; aaddch@gmail.com; 5 Institute of Analytical and Environmental Sciences, National Tsing Hua University, Hsinchu 30013, Taiwan; chouhc@mx.nthu.edu.tw; 6 Department of Life Sciences, Institute of Biomedical Science, National Chung Hsing University, Taichung 402, Taiwan; lincc@dragon.nchu.edu.tw; 7 Institute of Bioinformatics and Structural Biology and Department of Medical Sciences, National Tsing Hua University, Hsinchu 30013, Taiwan

**Keywords:** *Antrodia cinnamomea*, Leptin-deficient (*ob/ob*) mice, Anti-obesity, Intestinal Barrier, Hyperphagia

## Abstract

Obesity is associated with metabolic disorders. Thus, obesity prevention and treatment are essential for health. *Antrodia cinnamomea* (AC) is a multifunctional medicinal fungus used for the treatment of various diseases and for preventing diet-induced obesity. Leptin deficiency causes over-eating and spontaneous obesity. The concomitant metabolic symptoms are more severe than diet-induced obesity. Here, we used leptin-deficient (*ob/ob*) mice as an animal model for over-feeding to study the effect of AC on obesity. We fed C57BL/6 mice (WT, *ob+/+*) and *ob/ob* mice with AC for four weeks before performing qRT-PCR and immunoblot analysis to elaborate AC-modulated mechanisms. Further, we used Caco-2 cells as a human intestinal epithelial barrier model to examine the effect of AC on intestinal permeability. Our results suggested that AC reduces lipid deposits of the liver and epididymal white adipose tissue (EWAT) by promoting lipid metabolism and inhibiting lipogenesis-associated genes and proteins in *ob/ob* mice. Moreover, AC effectively repaired intestinal-barrier injury caused by leptin deficiency and enhanced intestinal barrier integrity in Caco-2 cells. Interestingly, AC significantly reduced body weight and EWAT with no compromise on food intake in *ob/ob* mice. Thus, AC effectively reduced obesity caused by leptin-deficiency and can potentially be used as a nutraceutical for treating obesity.

## 1. Introduction

Obesity refers to the state of being overweight, with abnormal fat accumulation on the body. Obesity is associated with multiple diseases like diabetes, certain cancers, hypertension, and cardiovascular diseases [1]. Obesity has been thought to be caused by personal habits, primarily excess calorie intake and lack of exercise. Furthermore, genetic components such as hereditary deficiency and genetic variations including leptin gene mutations could also have a strong effect on the development of excess weight gain and obesity [2,3,4]. Leptin is a hormone, primarily secreted by the adipose tissue, and affects the hypothalamus, regulates appetite and energy balance, and consequently, body weight gain [5,6]. Leptin deficiency (*Lep^ob^*; commonly called *ob* or *ob/ob*) causes spontaneous obesity in mice, resulting in *ob/ob* mice reaching a bodyweight thrice that of normal mice. The *ob/ob* mice exhibit symptoms such as hyperphagia, elevated plasma insulin, glucose intolerance, type II diabetes, and liver steatosis [7]. Many studies have used high fat-diet induced obese (DIO) mice and leptin-deficient (*ob/ob*) mice to mimic human obesity [8]. Both are excess energy uptake-related obesity and are similar in many aspects [9]. However, compared to DIO mice, *ob/ob* mice exhibit unregulated feeding (hyperphagia), which leads to obesity, diabetes, and disorders that are more severe than those seen in DIO mice [10].

Previously, *Antrodia cinnamomea* (AC) has been shown to prevent obesity and the development of fatty liver and regulate gut microbiota in DIO mice [11,12]. AC is a native medicinal fungus of Taiwan that only grows on the rotting heartwood of *Cinnamomum kanehirae* Hayata, a type of evergreen arbor [13]. AC is cultured using segment wood culture, liquid culture, or solid-state culture. The fruiting bodies and mycelia of AC contain polysaccharides, triterpenoids, steroids, benzenoids, benzoquinone derivates, phenolic compounds, cordycepin, sesquiterpene, adenosine, ergosterol, maleic/succinic acid derivatives, and other compounds [14]. AC has been widely used as a health remedy to prevent or treat various diseases like alcoholic liver disease, inflammation, and hypertension in Taiwan. AC also has the potential for preventing cancer, diabetes, and inflammatory diseases [11,15]. Aqueous AC extracts inhibit pre-adipocyte differentiation and adipogenesis in 3T3-L1 [16]. Notably, the previous study also demonstrated that the water extract of AC regulated the composition of the gut microbiota by decreasing the Firmicutes/Bacteroidetes ratio and increasing the abundance of *Akkermansia muciniphila* and other bacterial species associated with anti-inflammatory properties in DIO mice. Therefore, supplementation with AC reduces obesogenic and inflammatory effects in DIO mice by maintaining intestinal integrity and regulating the gut microbiota [11]. However, it is unclear whether AC protects or attenuates disease in obesity-related leptin-deficient (*ob/ob*) mice. Here, we used leptin-deficient (*ob/ob*) mice as an animal model for over-feeding to evaluate the potential of AC in anti-obesity roles in leptin-deficient (*ob/ob*) mice. We further investigated the role of AC in intestinal permeability and barrier integrity in Caco-2 cells.

## 2. Materials and Methods

### 2.1. Chemicals and Reagents

AC was obtained from DA-FU Agricultural Biotech Co., Ltd., Hsinchu City, Taiwan. Commonly used chemicals were purchased from Sigma-Aldrich (St. Louis, MO, USA). All primary antibodies were purchased from GeneTex (Hsinchu City, Taiwan) and secondary antibodies were purchased from GE Healthcare (Uppsala, Sweden).

### 2.2. Mice

B6.V-Lep^ob^/JNarl *(ob/+)* mice were obtained from the National Laboratory Animal Center and maintained in National Health Research Institutes (NHRI). Three to four mice were kept per cage under a 12 h light cycle and fed an autoclaved chow diet (1324 M, altromin, Lage, Germany). For AC treatment, mice were orally gavaged with 1.6667 g AC per kg body weight. AC was resuspended in phosphate-buffered saline (PBS), and 300 μL of AC or PBS (vehicle control) was administered thrice a week for 4 weeks [15,17]. The mice were assigned to four groups (8–14 mice per group)—*ob+/+*-Ctrl, *ob+/+*-AC, *ob/ob*-Ctrl, and *ob/ob*-AC. To mimic treatment on early-stages and exclude downregulated metabolism by aging, the experiment was started in 4–5-week-old male mice and the mice were euthanized after a 4-week treatment period; the experiment was performed as previously described [18], with some modifications. Each group comprised 4–5-week-old male mice of similar initial body weight (no blinding). Body weight was monitored every week from the day before the first time (T0) to the day after the 4-week (T4) treatment (with AC or PBS) period; daily food and water intake and fecal and urine weight were recorded at T0 and T4 using a metabolic cage. All animal experiments were performed according to the protocols approved by the institutional animal care and use committee of the National Health Research Institutes (Approval No. NHRI-IACUC-107046-A). All experiments were performed following the guidelines.

### 2.3. Sample Collection and Histological Observation

The mice were euthanized using carbon dioxide overdose. The liver, intestine, and epididymal white adipose tissue (EWAT) were harvested, fixed in 4% formaldehyde, paraffin-embedded, sectioned, and stained using hematoxylin and eosin (H&E) or immunohistochemistry (IHC). IHC was performed as previously described [19]. The appropriate volume of primary anti-cluster of differentiation 36 (CD36) (1:100, GTX100642, GeneTex) antibody was added to cover the specimen and the samples were incubated at 4 °C overnight. Nuclei were stained with hematoxylin. The images were captured using Pannoramic MIDI II (3DHISTECH Ltd., Budapest, Hungary).

### 2.4. RNA Extraction and Real Time RT-PCR

For real time RT-PCR, tissues were collected in *RNAlater* RNA Stabilization Reagent (QIAGEN, Hilden, Germany), snap-frozen in liquid nitrogen and stored at –80 °C. Total RNA was isolated from 50–100 mg of homogenized liver, intestine, and EWAT using the TRIzol reagent (ThermoFisher, Waltham, Massachusetts), as described previously [20]. cDNA was synthesized from 2 µg total RNA using M-MLV reverse transcriptase (Promega, Madison, WI, USA). Real-Time PCR reactions were performed on a LightCycler 480 System (Roche) and FastStart Universal SYBR Green Master (Rox) (Roche Diagnostics GmbH, Mannheim, Germany) was used for the reactions. Relative quantification was performed using the comparative 2^–ΔΔ^CT method [11]. The RNA expression profiles from liver, intestine, and EWAT were normalized to the 18S ribosome, TBP1, and HPRT respectively [15]. For detailed information of primers used in this study, see Supplementary Table S1.

### 2.5. Immunoblotting

For immunoblotting, tissues were collected, snap-frozen in liquid nitrogen, and stored at −80 °C. The tissues were homogenized and lysed in 1% Nonidet P40 Substitute lysis solution in the presence of 0.1% protease inhibitor. Immunoblotting was performed as previously described [21]. A total of 40–60 μg of protein samples were separated on a 12% 1D-SDS-PAGE and transferred to polyvinylidene difluoride membranes (Pall Corp., Port Washington, NY, USA). The membranes were blocked with 5% (w/v) skim milk or bovine serum albumin in Tris-buffered saline with Tween-20 (TBST; 50 mM Tris, 150 mM NaCl, and 0.1% Tween-20 (v/v); pH 8.0) for 1 h. Thereafter, the membranes were probed with the following primary antibodies (all from GeneTex): anti-acetyl-CoA carboxylase (ACC) (1:2000, GTX132081); anti-fatty acid synthase (FAS) (1:2000, GTX109833); anti- HMG-CoA reductase (HMGCR) (1:2000, GTX54088); anti-cytosolic malic enzyme 1 (ME1) (1:2000, GTX104122); anti-carnitine palmitoyltransferase 1 (CPT1A) (1:2000, GTX114337); anti-fatty acid-CoA ligase 4 (FACL4) (1:2000, GTX114399); anti-fructose-1,6-bisphosphatase 1 (FBP1) (1:2000, GTX54007); anti-adipose triglyceride lipase (ATGL) (1:2000, GTX109941); anti-fatty acid binding protein 4 (FABP4) (1:2000, GTX116036); and anti-β-actin (1:20000, GTX109639) overnight at 4 °C. Subsequently, the membranes were washed in TBST (4 × 10 min) and incubated with horseradish peroxidase-coupled secondary antibodies (Jackson ImmunoResearch Laboratories, Inc., Baltimore, PA, USA) in TBST for 1 h. The membranes were then washed in TBST (6 × 10 min), and the immunoprobed proteins were visualized using the enhanced chemiluminescence method (Visual Protein Biotech Corp., Taiwan). Protein expression was quantified using the ImageQuant TL Software (GE Healthcare Life Sciences, Pittsburgh, PA, USA) and was normalized to that of β-actin, which was used as the internal control.

### 2.6. Cell Culture and Cell Viability

Caco-2 cells were cultured in Dulbecco’s modified Eagle’s medium, supplemented with 20% (*v*/*v*) fetal calf serum, L-glutamine (2 mM), penicillin (100 IU/mL), and streptomycin (100 μg/mL) (all from Gibco-Invitrogen Corp., UK) and incubated at 37 °C in 5% CO_2_. The cell viability assay was performed as described previously [21]. Briefly, Caco-2 cells (2 × 10^3^ per well) were seeded on 96-well plates for 48 h and treated with 0–3000 μL/mL ethanol extract of AC (EEAC) for 48 h. Ethanol extraction of *A. cinnamomea* was performed as described previously [22].

### 2.7. Transepithelial Electrical Resistance (TEER) 

Caco-2 cells were cultured and differentiated, as described previously [15]. Briefly, 1 × 10^4^ cells were seeded on a polyester membrane insert (Costar 3470, Corning). Medium, with or without EEAC (500 μg/mL), was changed thrice a week. TEER was measured once a week for 3 weeks using a Voltohmmeter (Millicell ERS-2, Millipore) [23].

### 2.8. Immunofluorescence

Caco-2 cells were seeded and treated with or without EEAC (500 μg/mL) for 72 h. Thereafter, the cells were fixed in 4% formaldehyde for 5 min, washed with PBS, blocked with 1% BSA/PBS/1% FBS for 1 h, and incubated with the zona occludens-1 (ZO-1) antibody (1:50; 40–2300, Thermo Scientific) overnight at 4 °C. Subsequently, the cells were washed with PBS and incubated with donkey anti-goat IgG (1:400; A11055, Thermo Scientific) at room temperature for 1 h. Finally, the cells on the coverslips were stained with 4’,6-diamidino-2-phenylindole (DAPI) for 10 min and washed with PBS once for 5 min. Images were captured using the Leica DM2500 microscope [15]. Caco-2 cells treated without EEAC and anti-ZO-1 were used as negative controls.

### 2.9. Statistical Analysis

All statistical analyses were performed using GraphPad Software of Prism 6.0. Comparisons between two and more than two groups were done using the unpaired T test and two-way ANOVA, followed by Tukey’s multiple comparisons test. Data are presented as mean ± SEM. Statistical significance levels are indicated as * *p* < 0.05, ** *p* < 0.01, and *** *p* < 0.001; non-significant comparisons are marked as ns.

## 3. Results

### 3.1. AC has an Anti-obesity Effect in ob/ob Mice

**Figure 1:**
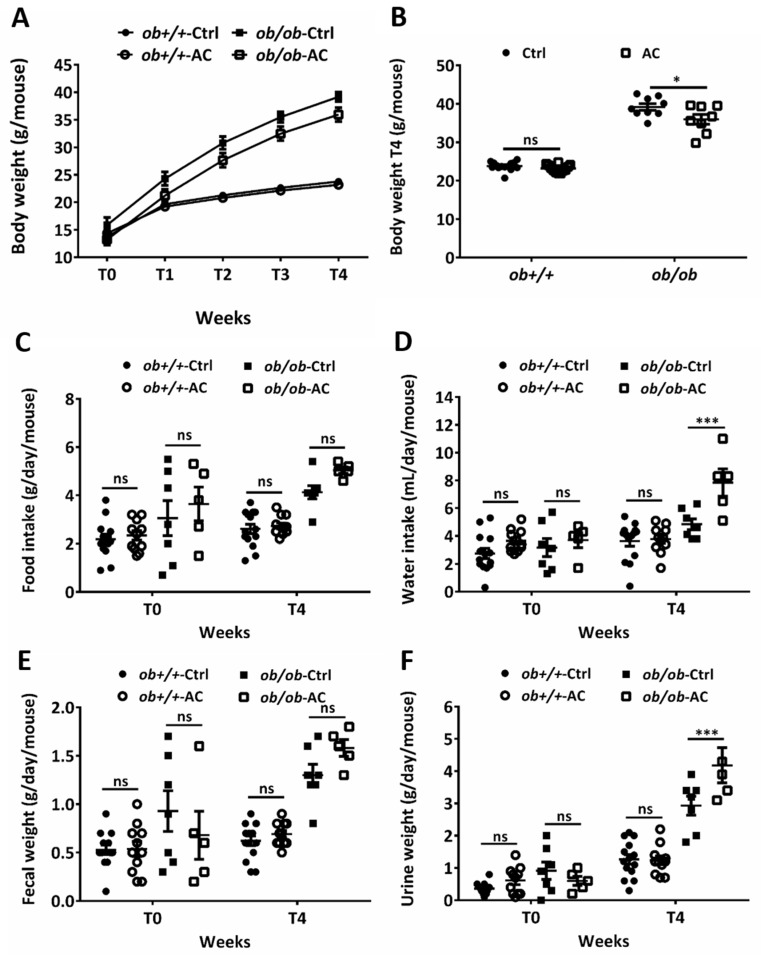
AC reduces body weight in *ob/ob* mice. (**A**) Body weights of 4–5 week-old *ob+/+* and *ob/ob* fed a diet with or without AC were measured every week for 4 weeks. (**B**) Body weights of *ob+/+* and *ob/ob* fed with or without AC were measured after 4 weeks (T4). (**C**–**F**) Daily food or water intake and fecal or urine weights were monitored using a metabolic cage at T0 and T4. All data are expressed as mean ± SEM, * *p* < 0.05, ** *p* < 0.01, *** *p* < 0.001. Non-significant; ns. *n* = 5–14 mice in each group.

To understand the effect of AC on overfeeding-induced obesity, *ob+/+* and *ob/ob* mice were fed with AC thrice a week for four weeks and their body weight was measured every week. The experiment was started in 4–5-week-old mice before spontaneous obesity developed [24]. After four weeks of treatment, the average body weights in *ob+/+* mice, fed without or with AC, were 23.73 g and 23.17 g, respectively; 39.17 g and 35.95 g in *ob/ob* mice fed without or with AC, respectively (Figure 1A). AC significantly reduced the 8% weight gain in *ob/ob* mice compared to *ob/ob* mice fed without AC (Figure 1B). Simultaneously, AC significantly increased daily water intake and urine weight in *ob/ob* mice; however, food intake and fecal weight remained unchanged in *ob+/+* mice (Figure 1C–F). These results suggested that AC inhibits the obesity phenotype of *ob/ob* mice without any compromise on food consumption and defecation.

### 3.2. AC Alleviates Hepatic Lipid Accumulation and Lipid Deposition in EWAT in ob+/+ and ob/ob Mice

**Figure 2:**
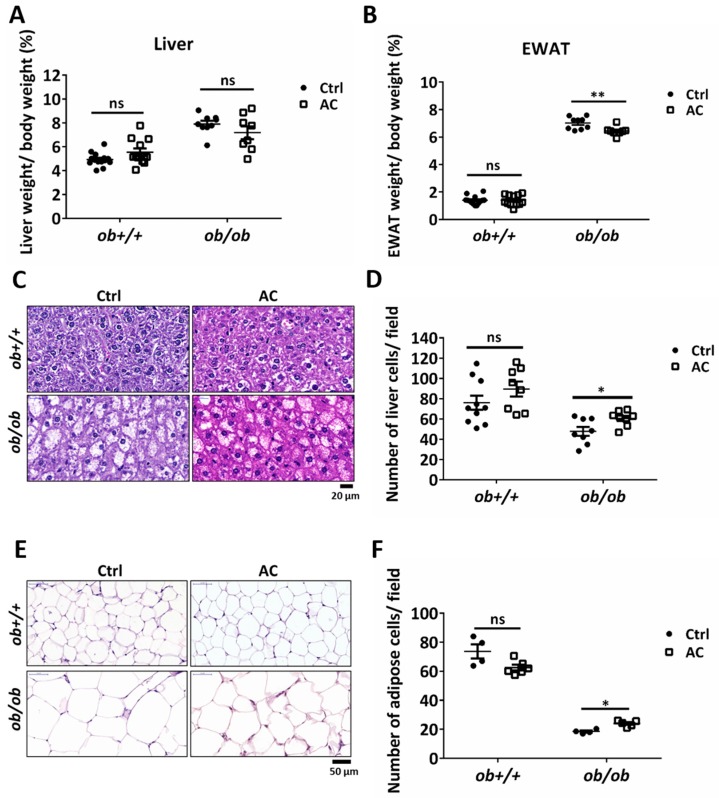
AC may suppress lipid accumulation and deposition in the liver and EWAT in *ob/ob* mice. (**A**,**B**) Percentage of liver or epididymal white adipose tissue (EWAT) weight were normalized to body weight after *ob+/+* and *ob/ob* mice were fed with or without AC for 4 weeks. (**C**,**E**) The liver and EWAT were examined using hematoxylin and eosin staining. (**D**,**F**) The number of liver or EWAT cells per field was estimated using the ImageJ software. Magnification, 100×. Scale bars are 20 μm for the liver and 50 μm for EWAT. All data are expressed as mean ± SEM, * *p* < 0.05, ** *p* < 0.01, *** *p* < 0.001. Non-significant; ns. *n* = 4–14 mice in each group.

We examined the effect of AC on the liver and EWAT of *ob/ob* mice after a four-week stimulation period. Although liver weight remained unaltered, EWAT weight was significantly reduced in *ob/ob* mice (Figure 2A,B). We also used H&E staining to examine the degree of lipid content in liver cells and EWAT. The number of cells in the liver and EWAT was evaluated using the number of nuclei or cells per field. We observed a change in lipid drop size in the cells. AC significantly reduced hepatic lipid content and EWAT fat deposition in *ob/ob* mice (Figure 2C–F). Here, we found that AC could reduce or prevent lipid accumulation in the liver and lipid deposition in the EWAT.

### 3.3. AC Downregulates Fatty Acid Uptake and Lipogenesis-associated Genes and Proteins in the Liver of ob+/+ and ob/ob Mice

**Figure 3:**
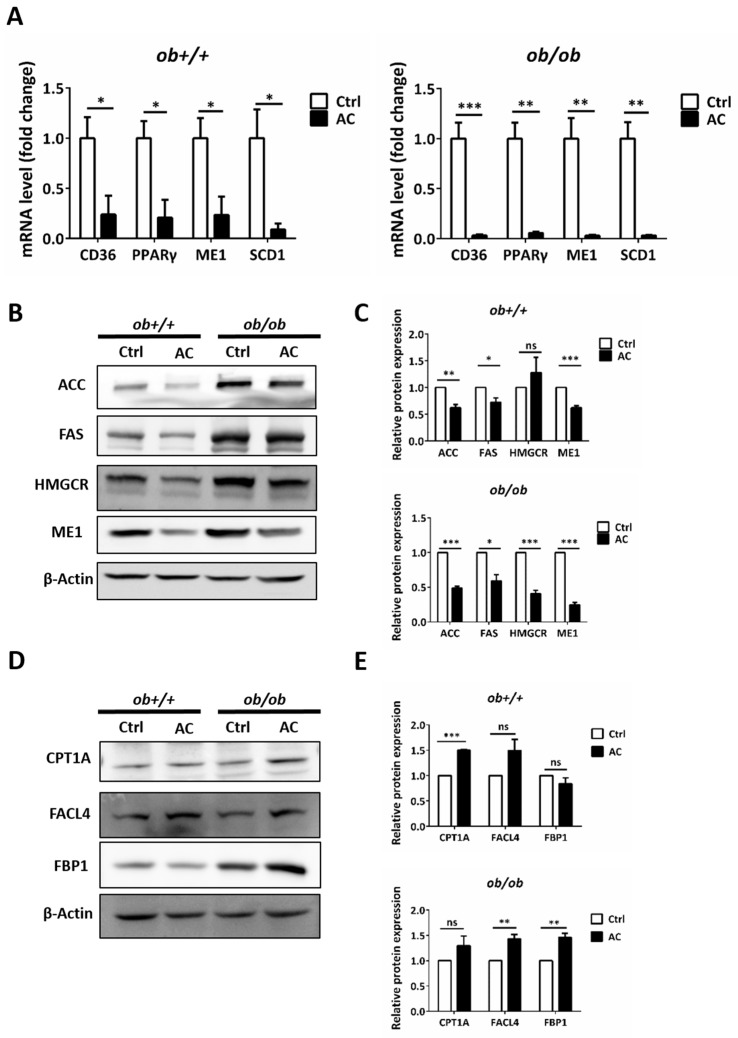
AC reduces hepatic lipogenesis and increases gluconeogenesis by regulating related-genes or protein in *ob+/+* and *ob/ob* mice. (**A**) The relative mRNA expression of CD36, PPARγ, ME1, and SCD1 were compared between *ob+/+*Ctrl or *ob*/*ob*-Ctrl mice. (**B**,**D**) Immunoblot analysis of proteins involved in lipogenesis, β-oxidation, and gluconeogenesis. (**C**,**E**) Quantification of protein expression and comparison between *ob+/+*-Ctrl and *ob/ob*-Ctrl mice. All data are expressed as mean ± SEM, * *p* < 0.05, ** *p* < 0.01, *** *p* < 0.001. Non-significant; ns. *n* = 4–8 mice in each group. All experiments were repeated thrice.

To investigate how AC improves lipid accumulation in *ob/ob* mice, we used real time RT-PCR to evaluate regulation of expression of genes involved in lipid catabolism and lipogenesis by AC. We found that AC significantly suppressed the gene expression of peroxisome proliferator-activated receptor gamma (PPARγ), CD36, ME1, and SCD1 in *ob+/+* and *ob/ob* mice (Figure 3A). In addition, a comparison of *ob+/+*-Ctrl and *ob/ob*-AC results showed that AC could restore lipid catabolism and lipogenesis-related gene expression to the normal level in *ob/ob* mice. Next, we used immunoblotting to further clarify the mechanism. AC significantly decreased expression of fatty acid synthesis-related proteins, such as ACC, FAS, and HMGCR in *ob+/+* and *ob/ob* mice (Figure 3B,C). In addition, AC treatment appeared to increase fatty acid β-oxidation in the mitochondria and peroxisomes, as seen by the up-regulation of CPT1A and FACL4, respectively (Figure 3D,E). Notably, AC promoted gluconeogenesis by increasing FBP1 expression and decreasing lipid accumulation (Figure 3D,E). These results demonstrated that AC could prevent lipid accumulation in the liver of *ob/ob* mice by reducing the expression, at the mRNA and protein level, of genes involved in lipid uptake and lipogenesis, as well as promoting the expression, at the mRNA and protein level, of genes involved in lipid catabolism.

### 3.4. AC Promotes Lipolysis-associated Protein Expression in EWAT of ob/ob Mice

**Figure 4:**
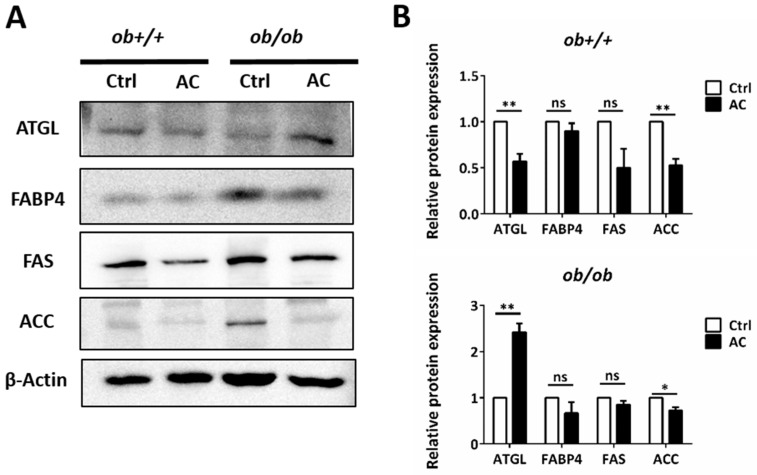
AC promotes expression of a lipolysis-associated protein in the EWAT of *ob+/+* and *ob/ob* mice. (**A**) Immunoblot analysis of lipolysis pathway protein. (**B**) Quantification and comparison of protein expression in *ob+/+*-Ctrl or *ob/ob*-Ctrl mice. All data are expressed as mean ± SEM, * *p* < 0.05, ** *p* < 0.01, *** *p* < 0.001. Non-significant; ns. *n* = 4–8 mice in each group. All experiments were repeated thrice.

We used H&E staining to show that lipid depositions were significantly reduced by AC in the EWAT of *ob/ob* mice. To investigate the effects of AC in the EWAT of *ob+/+* and *ob/ob* mice, we used immunoblotting. The protein of ACC expression was reduced in the EWAT of *ob/ob* mice fed with AC, suggesting that AC suppressed lipogenesis (Figure 4A,B). Notably, the protein levels of ATGL, a lipid droplet degradation (lipolysis) protein, were increased 2.4 times in the EWAT of *ob/ob* mice, fed with AC, after four weeks. ATGL played a key role in the EWAT by decreasing lipid accumulation (Figure 4A,B). These results suggested that AC down-regulated lipogenesis and up-regulated a lipolysis-associated protein to decrease fat deposition in the EWAT of *ob/ob* mice.

### 3.5. AC may Restore the Intestinal Barrier in ob/ob Mice

**Figure 5:**
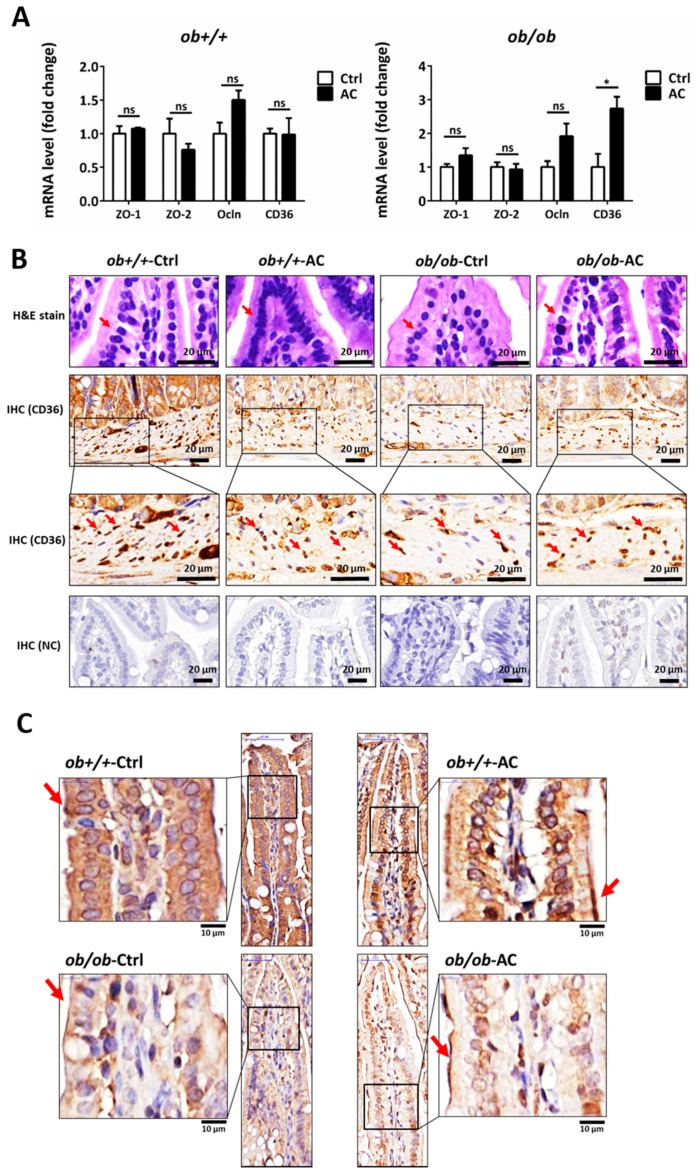
The intestinal barrier is maintained by AC in *ob/ob* mice. (**A**) Relative mRNA expression of tight junction proteins and CD36 between *ob+/+*-Ctrl and *ob/ob*-Ctrl mice; (**B**) The intestine was examined using hematoxylin and eosin staining and immunohistochemical staining of CD36. Magnification, 40×. Scale bars are 20 μm. The negative control for immunohistochemistry has been marked NC; (**C**) The intestine was examined using immunohistochemical staining for ZO-1. Magnification, 40× and 100×; scale bars are 50 μm and 10 μm, respectively. All data are expressed as mean ± SEM, * *p* < 0.05, ** *p* < 0.01, *** *p* < 0.001. Non-significant; ns. *n* = 4–8 mice in each group.

The intestine is at the front line of absorbing nutrients and lipids and the intestinal barrier integrity and permeability are thought to be involved in certain chronic inflammatory diseases such as inflammatory bowel disease (IBD), obesity, and other metabolic disorders [25]. Previously, AC was shown to produce an anti-obesity and anti-inflammatory effect by maintaining intestinal integrity in DIO mice; moreover, CD36 deletion in endothelial cells of the small intestine resulted in impaired barrier function of the small intestinal in mice [11,26]. We found that CD36 expression was restored in the small intestine of *ob/ob* mice and unaffected in *ob+/+* mice, after AC treatment (Figure 5A). Levels of tight junction proteins, like zonula occludens-1 (ZO-1) and zonula occludens-2 (ZO-2), that maintain intestinal permeability were unchanged. However, the level of occludin (Ocln) was slightly increased in *ob+/+* or *ob/ob* mice fed with AC, after four weeks (Figure 5A). Moreover, H&E staining showed increased intestinal barrier integrity in *ob/ob* mice fed with AC than *ob/ob* mice not fed AC (Figure 5B). We also examined CD36 and ZO-1 localization in the intestine using IHC. AC restored CD36 expression in endothelial cells (Figure 5B). Moreover, AC promoted ZO-1 localization in intestinal epithelial cells in *ob/ob* mice compared to *ob/ob-*Ctrl (Figure 5C). Thus, AC repairs the intestinal barrier by up-regulating CD36 expression, redistributing ZO-1, and reducing intestinal permeability in *ob+/+* and *ob/ob* mice.

### 3.6. Ethanol Extracts of A. Cinnamomea Decrease Intestinal Permeability in Caco-2 Cells

**Figure 6:**
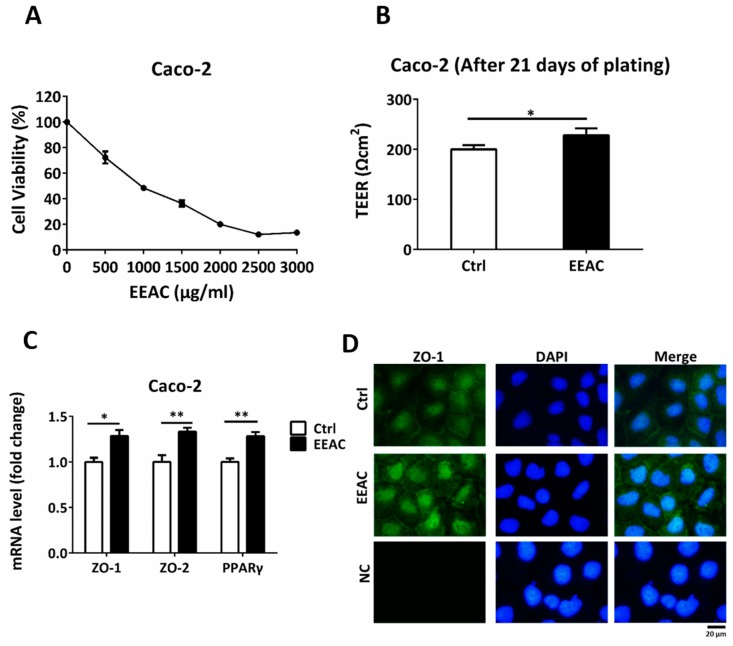
EEAC decreases intestinal permeability in the Caco-2 cell barrier model. (**A**) Cell viability assay of Caco-2 cells treated with 0-3000 μL/mL EEAC. (**B**) Effect of EEAC on intestinal permeability was measured using the trans-epithelial electrical resistance (TEER) assay. (**C**) Relative mRNA expression of tight junction proteins and PPARγ were compared using controls (Ctrl). (**D**) Immunofluorescence staining of ZO-1 (green) of Caco-2 cells treated with or without EEAC. Nuclei were stained with DAPI (blue). The negative control is marked as NC. Scale bars, 20 μm. All data are expressed as mean ± SEM, * *p* < 0.05, ** *p* < 0.01, *** *p* < 0.001. Non-significant; ns. All experiments were repeated thrice (*n* = 3).

To further examine the effect of AC on the human intestine, we used Caco-2 cells, as a human intestinal epithelial cell barrier model, treated with 500 μL/mL EEAC, according to the cell viability assay (IC25), to understand whether EEAC affected intestinal permeability (Figure 6A) [23]. Integrity of the Caco-2 membrane was assessed using TEER values after cell seeding for 7, 14, and 21 days. After 21 days, the TEER values of the Caco-2 membrane, treated with EEAC, were significantly higher than that of the control group (Ctrl) (Figure 6B). EEAC increased the gene of PPARγ expression and induced the upregulation of tight junction proteins, including ZO-1 and ZO-2, in Caco-2 cells (Figure 6C). Moreover, ZO-1 levels were enhanced in Caco-2 cells treated with EEAC (Figure 6D). These results suggested that AC enhanced intestinal barrier integrity and decreased intestinal permeability.

Thus, AC administration inhibited hepatic lipogenesis and lipid uptake; promoted lipolysis and reduced lipogenesis to prevent fat deposition in the EWAT in *ob/ob* mice. In addition, AC restored intestinal barrier integrity in *ob/ob* mice, enhanced intestinal barrier integrity, and decreased intestinal permeability in Caco-2 cells. Our study provides a rationale for the anti-obesity effect and intestinal protection effect of AC in leptin-deficient obese mice.

## 4. Discussion

Leptin maintains the physiological balance of energy. It has an impact on metabolism and body weight and plays a key role in promoting body fat degradation [27]. Leptin secretions are regulated by factors such as excess energy stored as fat, overfeeding, glucose and insulin levels, and inflammatory cytokines [28]. Leptin deficiency could cause symptoms including early-onset morbid obesity, hyperphagia, hypogonadotropic hypogonadism, advanced bone age, hyperinsulinemia, and immune dysfunction [28]. The previous studies have used Roux-en-Y gastric bypass surgery, food or calorie restriction, leptin administration, and adipose tissue transplantation to treat leptin-deficient mice [9,29,30,31]. Currently, metreleptin (Myalept), a recombinant human leptin analog, is used as an injectible to treat complications of leptin deficiency in patients with congenital or acquired generalized lipodystrophy. Although metreleptin was approved by the Food and Drug Administration in 2014, it has common side effects like headache, ovarian cysts, ear infection, high levels of protein in the urine, fever, and leptin resistance [32,33].

The leptin-deficient *ob/ob* mice and DIO mice exhibit over-feeding and excessive energy uptake-derived obesity, and they are prone to many diseases like nonalcoholic fatty liver disease (NAFLD), hyperphagia, and type II diabetes [34,35,36]. Interestingly, we found that AC decreased body weight and lipid accumulation in the liver and EWAT, but it did not significantly affect food intake. Although a previous study showed that AC prevents obesity and fatty liver in DIO by regulating AMPK and SREBP signaling, here, the AMKP and SREBP signaling pathways were not affected by AC in *ob/ob* mice [12]. We further examined fatty acid uptake, lipogenesis, and the lipid catabolism pathway. PPARγ is a lipogenesis-related protein that has been shown to regulate lipid uptake, lipogenesis, and lipid storage. CD36 is an integral membrane protein, also called fatty acid translocase, that is involved in translocation of long-chain fatty acids [37,38]. Our results suggested that AC suppressed PPARγ and CD36 gene expression and reduced fatty acid transportation. Simultaneously, AC inhibited lipogenesis by decreasing the expression of ME1, which generates NADPH used for lipogenesis in the liver and adipose tissues, and SCD1, which is involved in fatty acid synthesis in the liver [39,40]. Moreover, ACC and FAS have been shown to be involved in fatty acid synthesis and HMGCR in the cholesterol synthesis pathway [41,42]. Here, our immunoblotting results showed that AC significantly inhibited lipogenesis by decreasing protein expression of ACC, FAS, and HMGCR in *ob+/+* and *ob/ob* mice. AC also promoted fatty acid β-oxidation in the mitochondria and peroxisomes by increasing protein expression of CPT1A and FACL4, which are the key enzymes that catalyze mitochondrial fatty acid oxidation in the liver [42,43]. Gluconeogenesis is a pathway of glucose metabolism that might assist to keep 3C substrates out of lipid metabolism, and synthesized glucose may be transported to other tissue. [44]. Our results showed that AC alleviated fatty liver mainly by decreasing fatty acid uptake (CD36), lipogenesis (PPARγ, SCD1, ACC, FAS, and ME1), and increasing gluconeogenesis (FBP1) in *ob/ob* mice. Simultaneously, AC also decreased lipid accumulation in the adipose tissue by decreasing lipogenesis (ACC) and facilitating lipolysis (ATGL) in *ob/ob* mice.

Intestinal barrier integrity and permeability are thought to contribute to inflammatory bowel disease, obesity, and metabolic disorders [45]. In previous studies, CD36 deletion in endothelial cells of the small intestine impaired the small intestinal barrier [26]. Therefore, CD36 influences lipid utilization, homeostasis, and barrier maintenance in the intestine, especially in intestinal endothelial cells [46]. Also, a previous study showed that AC can regulate gut microbiota and enhance antimicrobial peptide production [11]. Our results showed that AC restored the integrity of the intestinal barrier by increasing CD36 expression in endothelial cells and decreasing intestinal permeability in *ob/ob* mice. Further, tight junction proteins of the intestine are important in preventing the entry of harmful substances, such as microbial components, into the body [47]. Intestinal permeability is associated with various diseases and is a potential target for disease prevention and therapy [48]. A previous study has shown that AC regulates gut microbiota, prevents DIO, and decreases intestinal inflammation and obesity [11]. In our study, AC slightly increased the expression of intestinal Ocln gene and redistributed ZO-1 to the membrane in *ob/ob* mice. These results suggested that AC reduces obesity by regulating intestinal permeability and barrier integrity.

Caco-2 human intestinal epithelial cells, used as a gut barrier model, when treated with EEAC also demonstrated that AC could decrease intestinal permeability and reinforce intestinal barrier integrity by regulating ZO-1 expression on the membrane. Taken together, these results suggested that AC alleviates leptin-deficiency induced obesity and disorders by regulating lipid catabolism and restoring intestinal barrier integrity.

In conclusion, our results indicated that AC supplementation inhibited hepatic lipogenesis and lipid uptake in *ob/ob* mice. At the same time, AC promoted lipolysis and decrease lipogenesis to prevent fat deposition in the EWAT in *ob/ob* mice. Furthermore, AC enhanced intestinal barrier integrity as preventive protection in *ob/ob* mice. Our work provides evidence that AC supplementation effectively reduced leptin-deficiency-mediated obesity by regulating metabolism in the liver and EWAT and restoring the gut barrier integrity without any significant compromise on food intake. The AC extract could be potentially used as a nutraceutical for the treatment of obesity, and AC compounds could be further analyzed as potential targets for drug design.

## Supplementary Materials

The following are available online at https://www.mdpi.com/2072-6643/12/3/726/s1, Table S1: Primers for real time RT-PCR.
