# Supplementary material for: Antrodia cinnamomea Confers Obesity Resistance and Restores Intestinal Barrier Integrity in Leptin-deficient Obese Mice
Source: Nutrients. 2020 Mar 10;12(3):726. doi: 10.3390/nu12030726 (PMC7146579; doi:10.3390/nu12030726)
Supplement: Supplementary file 1 [file nutrients-12-00726-s001.pdf]

Table 1. Primers for real time RT-PCR.

Species	Gene	Direction	Sequence	Reference
Mus musculus	18S ribosome	Forward<br>Reverse	5'-gtaacccgttgaaccccatt-3'<br>5'-ccatccaatcggtagtagc-3'	[1]
Mus musculus	TBP1	Forward<br>Reverse	5'-caaacccagaattgttctcctt-3'<br>5'-atgtggtcttcctgaatccct-3'	[2]
Mus musculus	HPRT	Forward<br>Reverse	5'-gcctaagatgagcgcaagtt-3'<br>5'-tactaggcagatggccacagg-3'	[3]
Mus musculus	CD36	Forward<br>Reverse	5'-ggagcaactggtggatggtt-3'<br>5'-ttgagactctgaaaggatcagca-3'	[4]
Mus musculus	ME1	Forward<br>Reverse	5'-ctcataggagttgctgcaattgg-3'<br>5'-cgttgaaggcagccatatcc-3'	[4]
Mus musculus	PPARγ	Forward<br>Reverse	5'-gtgccagtttcgatccgtaga-3'<br>5'-ggccagcatcgtgtagatga-3'	[5]
Mus musculus	SCD1	Forward<br>Reverse	5'-ccgggagaatatcctggttt-3'<br>5'-cactggcagagtagtcgaagg-3'	[4]
Mus musculus	ZO-1	Forward<br>Reverse	5'-atgcagacccagcaaagg-3'<br>5'-tggttttgtctcatcatttcttca-3'	[6]
Mus musculus	ZO-2	Forward<br>Reverse	5'-ccgttcagcagcttaggaaa-3'<br>5'-gaaccttccggggtctctt-3'	This study
Mus musculus	Ocln	Forward<br>Reverse	5'-gtccgtgaggccttttga-3'<br>5'-ggtgcataatgattgggtttg-3'	[7]
Homo sapiens	TBP1	Forward<br>Reverse	5'-gaacatcatggatcagaacaaca-3'<br>5'-atagggattccgggagtcat-3'	[8]
Homo sapiens	ZO-1	Forward<br>Reverse	5'-caacatacagtgacgcttcaca-3'<br>5'-cactattgacgtttccccactc-3'	[9]
Homo sapiens	ZO-2	Forward<br>Reverse	5'-ggccaggttacagagaatgc-3'<br>5'-gcttctgggcaatttcgat-3'	[10]
Homo sapiens	PPARγ	Forward<br>Reverse	5'-ccattacggagagatccacg-3'<br>5'-aggccattttgtcaaacgag-3'	[11]

Reference

1. Rho, H.W., et al., Identification of valid reference genes for gene expression studies of human stomach cancer by reverse transcription-qPCR. BMC Cancer, 2010. **10**: p. 240.
2. Wang, F., et al., Normalizing genes for real-time polymerase chain reaction in epithelial and nonepithelial cells of mouse small intestine. Anal Biochem, 2010. **399**(2): p. 211-7.
3. Carrel, L., P.A. Hunt, and H.F. Willard, Tissue and lineage-specific variation in inactive X chromosome expression of the murine Smcx gene. Hum Mol Genet, 1996. **5**(9): p. 1361-6.
4. Jais, A., et al., Heme oxygenase-1 drives metaflammation and insulin resistance in mouse and man. Cell, 2014. **158**(1): p. 25-40.
5. Galmozzi, A., et al., ThermoMouse: an in vivo model to identify modulators of UCP1 expression in brown adipose tissue. Cell Rep, 2014. **9**(5): p. 1584-1593.
6. Ruan, J.W., et al., Dual-specificity phosphatase 6 deficiency regulates gut microbiome and transcriptome response against diet-induced obesity in mice. Nat Microbiol, 2016. **2**: p. 16220.
7. Kim, M.S. and J.Y. Kim, Ginger attenuates inflammation in a mouse model of dextran sulfate sodium-induced colitis. Food Sci Biotechnol, 2018. **27**(5): p. 1493-1501.
8. Hubner, M., et al., Intronic miR-744 Inhibits Glioblastoma Migration by Functionally Antagonizing Its Host Gene MAP2K4. Cancers (Basel), 2018. **10**(11).

9. Lv, J.W., et al., Inhibition of microRNA-214 promotes epithelial-mesenchymal transition process and induces interstitial cystitis in postmenopausal women by upregulating Mfn2. Exp Mol Med, 2017. **49**(7): p. e357.
10. Anderson, R.C., et al., Lactobacillus plantarum MB452 enhances the function of the intestinal barrier by increasing the expression levels of genes involved in tight junction formation. BMC Microbiol, 2010. **10**: p. 316.
11. Knarr, M., et al., miR-181a modulates circadian rhythm in immortalized bone marrow and adipose derived stromal cells and promotes differentiation through the regulation of PER3. Sci Rep, 2019. **9**(1): p. 307.

© 2020 by the authors. Licensee MDPI, Basel, Switzerland. This article is an open access article distributed under the terms and conditions of the Creative Commons Attribution (CC BY) license (http://creativecommons.org/licenses/by/4.0/).
